# A New Transcriptional Repressor of the *Pseudomonas aeruginosa* Quorum Sensing Receptor Gene *lasR*

**DOI:** 10.1371/journal.pone.0069554

**Published:** 2013-07-05

**Authors:** Francesca Longo, Giordano Rampioni, Roslen Bondì, Francesco Imperi, Gian Maria Fimia, Paolo Visca, Elisabetta Zennaro, Livia Leoni

**Affiliations:** 1 Department of Sciences, University Roma Tre, Rome, Italy; 2 Department of Biology and Biotechnology “Charles Darwin”, Sapienza University, Rome, Italy; 3 National Institute for Infectious Disease “Lazzaro Spallanzani”, Rome, Italy; Ghent University, Belgium

## Abstract

*Pseudomonas aeruginosa* pathogenic potential is controlled *via* multiple regulatory pathways, including three quorum sensing (QS) systems. LasR is a key QS signal receptor since it acts as a global transcriptional regulator required for optimal expression of main virulence factors. *P. aeruginosa* modulates the QS response by integrating this cell density-dependent circuit to environmental and metabolic cues. Hence, QS also controls the adaptation to challenging environmental niches, such as infection sites. However, little is known about the molecular mechanisms connecting QS and other signalling pathways. In this work, DNA-affinity chromatography was used to identify new *lasR* transcriptional regulators. This approach led to the identification and functional characterization of the TetR-like transcriptional repressor PA3699. This protein was purified and shown to directly bind to the *lasR* promoter region *in vitro*. The induction of PA3699 expression in *P. aeruginosa* PAO1 cultures repressed *lasR* promoter activity and the production of LasR-dependent virulence factors, such as elastase, pyocyanin, and proteases. These findings suggest a role for PA3699 in *P. aeruginosa* pathogenicity. *P. aeruginosa* genome encodes at least 38 TetR-family proteins, and PA3699 is the eighth member of this group functionally characterized so far and the first one shown to bind the *lasR* promoter *in vitro.*

## Introduction

The definition of quorum sensing (QS) was coined about 20 years ago to describe a communication system based on the production, detection, and response to signal molecules, allowing bacterial populations to trigger a coordinated response at a threshold cell density [1]. Today it is well known that QS regulation plays a key role in a number of relevant bacterial processes, including colonization of plants and animal tissues, and production of antibiotics [2,3].

In many bacteria, the QS regulatory device is interwoven with other global regulatory networks responsive to different environmental cues (e.g., temperature, pH, osmolarity, oxidative stress, nutrient starvation). These networks cross-talk in the bacterial cell in order to determine the optimal survival strategy [4]. The advantage of integrating QS with other regulatory pathways becomes a compelling necessity when the QS response controls multiple functions and its activation commits bacteria to a strong reorganization of the whole cellular metabolism [5].

One of the most studied model organisms in QS research is *Pseudomonas aeruginosa*. This versatile bacterium is able to thrive in a wide range of environmental niches, including the human body. The high adaptability of *P. aeruginosa* is reflected by its behaviour as a pathogen. In humans, *P. aeruginosa* causes community- and hospital-acquired infections, colonizing different body districts like lungs, eyes, ears, urinary tract, injured skin (burns and wounds). Such infections are often difficult to eradicate as a consequence of antibiotic resistance and biofilm formation [6]. In particular, *P. aeruginosa* chronic lung infection is the major cause of death in cystic fibrosis patients, a genetic disease affecting about 1/3,000 newborns in the Caucasian population [6].

*P. aeruginosa* pathogenicity strongly depends on the fine and coordinated regulation of a wide array of virulence factors [7,8]. This is achieved thanks to a complex network of regulatory and signalling pathways controlling virulence-related phenotypes, in response to environmental cues and bacterial population structure [9]. Among these pathways, a preeminent role in *P. aeruginosa* virulence is played by three interconnected QS systems, based on the production of different signal molecules. The QS signal molecule *N*-3-oxo-dodecanoyl-homoserine lactone (3OC_12_-HSL) is synthesized by LasI, encoded by the *lasI* gene, and its cognate receptor is the cytoplasmic transcriptional regulator LasR, encoded by the *lasR* gene. 3OC_12_-HSL progressively accumulates in the bacterial culture and at a threshold concentration it binds LasR. The LasR/3OC_12_-HSL complex, in turn, activates transcription of hundreds of genes, including the genes coding for the receptors of the other two QS systems, based on *N*-butanoylhomoserine lactone (C_4_-HSL) and 2-heptyl-3-hydroxy-4-quinolone (PQS) signal molecules. As a whole, the *P. aeruginosa* QS circuit has a key role in pathogenesis, regulating the production of virulence factors, the formation of biofilm and the expression of antibiotic efflux pumps [10].

The three QS systems of *P. aeruginosa* are interwoven and connected to other regulatory pathways [10–12]. Indeed the expression of genes involved in the synthesis and perception of QS signal molecules is finely regulated at the transcriptional and post-transcriptional level in response to various metabolic and environmental stimuli [12–15]. However, the proteins involved in this regulation and their mechanisms of action are largely unknown [11,12]. To date only few transcriptional regulators have been shown to directly bind the promoters of genes involved in *P. aeruginosa* QS signal synthesis and perception. In particular, RsaL directly represses *lasI* transcription [16,17], while Vfr directly activates the *lasR* promoter region [18]. Moreover, AlgR2 (AlgQ), besides acting as an anti-sigma factor for the vegetative sigma RpoD [19], can also bind to and downregulate the *lasR* promoter [20]. However, the stimuli controlling the transcriptional activity of Vfr, AlgR2 (AlgQ) and RsaL are still unknown.

The objective of this work has been the identification of novel transcriptional regulators of LasR expression. To this aim, *P. aeruginosa* cytoplasmic proteins able to bind the *lasR* promoter region have been picked-up by DNA-affinity chromatography and identified by mass spectrometry. This led to the functional characterization of the TetR-like protein PA3699, which acts as a novel repressor of *lasR* transcription and of *P. aeruginosa* virulence factors production.

## Results and Discussion

### Identification of *P. aeruginosa* proteins interacting with the *lasR* promoter

**Figure 1:**
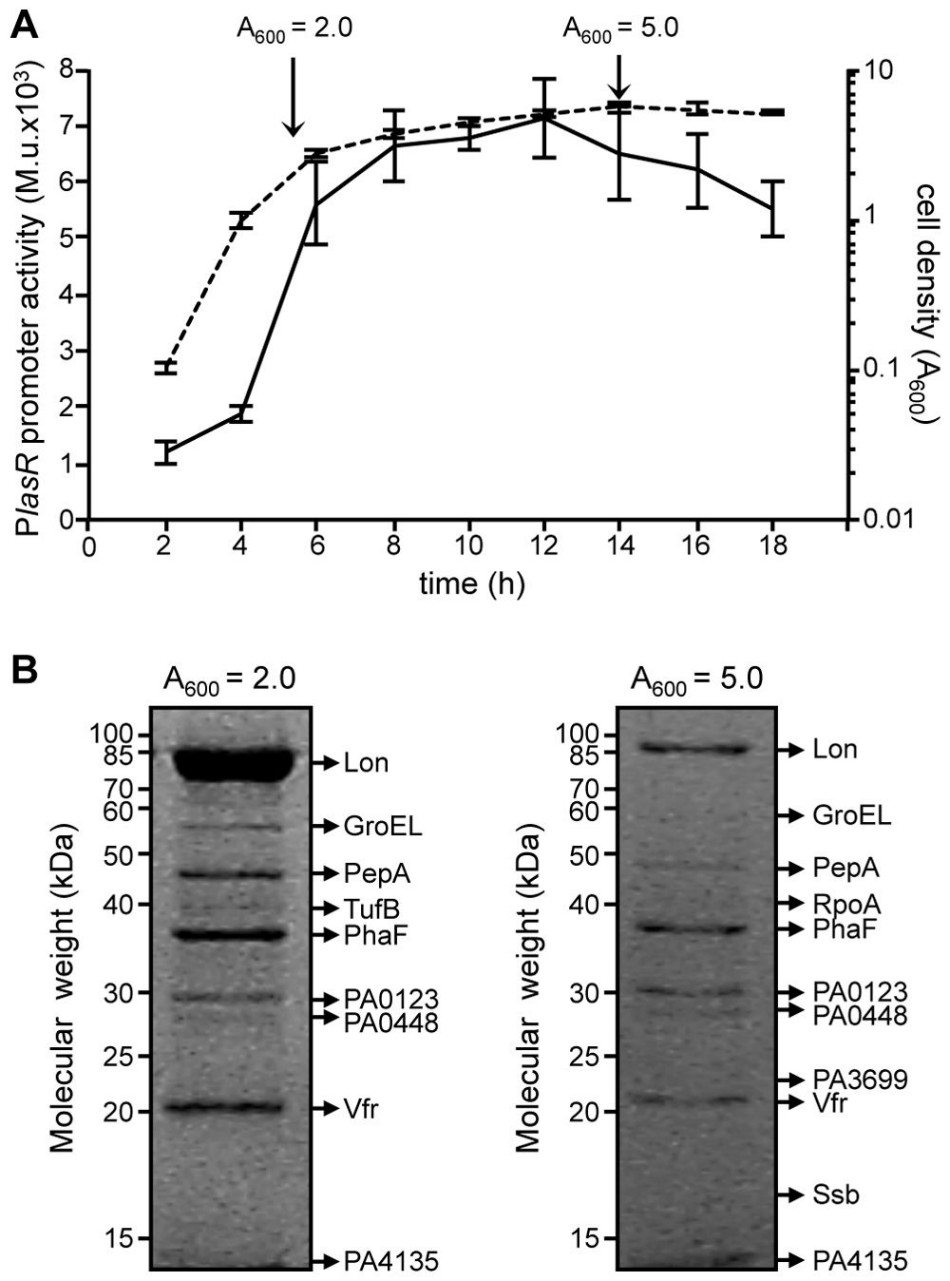
P*lasR* activity and P*lasR*-affinity chromatography. (A) Growth curve of the *P. aeruginosa* PAO1 wild type strain carrying the pMP*lasR*::*lacZ* plasmid (dashed line) and corresponding P*lasR* promoter activity (solid line). The points of the growth curve at which the protein crude extracts for DNA-affinity chromatography were prepared are indicated by arrows. (B) SDS-PAGE analysis of proteins bound to the P*lasR* promoter region. Protein crude extracts were prepared from *P. aeruginosa* PAO1 cultures grown in LB broth to the indicated cell densities (A_600_). Bands analysed by MALDI-TOF mass spectrometry are indicated by arrows, and the corresponding protein name is reported.

To increase the probability of fishing proteins interacting with the *lasR* promoter (P*lasR*), the activity of P*lasR* was preliminarily monitored along the *P. aeruginosa* PAO1 growth curve by means of a P*lasR*::*lacZ* transcriptional fusion (Figure 1A Table S2). P*lasR* activity increased steadily during the exponential phase and reached a plateau at the onset of the stationary phase. The maximal promoter activity was maintained for about four hours, and then declined (Figure 1A), suggesting that at the end of the exponential phase and during the stationary phase of growth, one or more transcriptional regulator(s) bind to P*lasR* and affect its promoter activity. Hence, crude protein extracts were prepared from *P. aeruginosa* PAO1 cultures grown to A_600_ = 2.0 and A_600_= 5.0, corresponding to late exponential and stationary phase of growth, respectively (Figure 1A).

To prepare the DNA-affinity chromatography matrix, a biotinylated double-stranded DNA fragment, corresponding to the *lasR* promoter region cloned in the P*lasR*::*lacZ* transcriptional fusion, was immobilized on a streptavidin-conjugated chromatography resin. Protein crude extracts were independently incubated with the matrix and, after extensive washing, proteins specifically bound to P*lasR* were eluted, separated by SDS-PAGE, and identified by MALDI-TOF mass spectrometry (Table 1). The experiment was performed in duplicate for each crude extract. Examples of SDS-PAGE gels are shown in Figure 1B. Overall, twelve protein bands were reproducibly detected on the SDS-PAGE gels (Table 1), while no protein bands were visible in control experiments with beads uncoupled to DNA (data not shown). Among these, RpoA, Ssb and TufB are involved in general DNA processing, while the PhaF phasin is involved in polyhydroxyalkanoate segregation during cell division [21]. Moreover three proteins not involved in DNA processing (i.e., Lon, PepA and GroEL) were also retrieved (Table 1).

**Table 1:** List of P*lasR*-binding proteins identified by MALDI-TOF analysis from the SDS-PAGE gels shown in Figure 1B.

**PA number ^a b^**	**Gene name^a^**	**Functional class^a^**	**Predicted molecular mass (kDa)**	**Protein score^*c*^ – No. of peptides matched**	
				A_600_ = 2.0	A_600_ = 5.0
**PA0123**		probable transcriptional regulator	33.5	447-10	708-21
**PA0448**		probable transcriptional regulator	34.1	652-18	777-19
**PA0652**	*vfr*	Transcriptional regulator Vfr	24.5	564-14	621-16
PA0779	*lon*	probable ATP-dependent protease	88.6	276-11	709-34
**PA3699**		probable transcriptional regulator	26.3	-	279-13
PA3831	*pepA*	leucine aminopeptidase	52.6	891-24	412-20
**PA4135**		probable transcriptional regulator	16.5	293-10	258-9
PA4232	*ssb*	single-stranded DNA-binding protein	18.5	-	134-6
PA4238	*rpoA*	DNA-directed RNA polymerase alpha chain	39.7	-	390-14
PA4277	*tufB*	elongation factor Tu	43.7	709-21	-
PA4385	*groEL*	GroEL protein	57.1	1210-22	723-21
PA5060	*phaF*	polyhydroxyalkanoate synthesis protein PhaF	30.6	439-11	456-20

a PA number, gene name and functional class refer to the *Pseudomonas* Genome Database annotation [22].

^b^ Proteins identified only when using P*lasR* as DNA bait are in bold characters.

c The protein score is according to the Mascot programme (scores ≥ 76 correspond to an error probability *p* < 0.05 in our data set) [37].

Of the two transcription factors previously known to bind P*lasR* (i.e., Vfr and AlgR2) [18,20], only Vfr was picked-up (Table 1), indicating that the DNA-affinity chromatography approach here described allows identification of some, but not all, the transcription factors specific for P*lasR*. Therefore, besides AlgR2, other regulators of this promoter may have escaped our analysis.

Last, but of primary importance with respect to our aims, among the picked-up proteins PA0123, PA0448, PA3699 and PA4135 were annotated in the *P. aeruginosa* genome as putative transcription factors with unknown function (Table 1). On the basis of their sequence, PA0123 and PA0448 are annotated as putative LysR-like family transcriprional regulators [22]. Interestingly, the three-dimensional structure of PA3699 and PA4135 has been recently solved (Protein Data Bank accession numbers, 3KKD and 2FBI, respectively). On the basis of primary sequence and structural features, PA3699 and PA4135 can be assigned to the TetR-like and MarR-like family of transcriptional regulators, respectively [22]. In this view, the above results provide the first experimental evidence that PA0123, PA0448, PA3699 and PA4135 are expressed in *P. aeruginosa* cultures and able to bind DNA *in vitro*. On the basis of the above considerations, these four factors were selected for further analysis as novel putative *lasR* regulators (Table 1).

### *In vivo characterization* of putative *lasR* transcriptional regulators

In order to study the *in vivo* effect of PA0123, PA0448, PA3699 and PA4135 on P*lasR* activity, a set of *P. aeruginosa* PAO1 in frame deletion mutants in the corresponding genes was generated. As a control, also a mutation in the *vfr* gene was introduced in *P. aeruginosa* PAO1. A genetic cassette carrying the P*lasR*::*lux* transcriptional fusion was introduced in a chromosomal neutral site of the five mutant strains and of *P. aeruginosa* wild type to conveniently measure P*lasR* activity as a function of light emission along the growth curve. The DNA region cloned in this genetic cassette was identical to that used as a bait in the DNA-affinity chromatography experiment.

In accordance with literature data, P*lasR* activity was strongly decreased as a consequence of *vfr* mutation (Figure S1) [18], while no significant differences with respect to the wild type activity were observed in the PA0123, PA0448, PA3699 and PA4135 mutant strains (Figure S1). Similar results were obtained by using the P*lasR*::*lacZ* transcriptional fusion as reporter system (data not shown).

**Figure 2:**
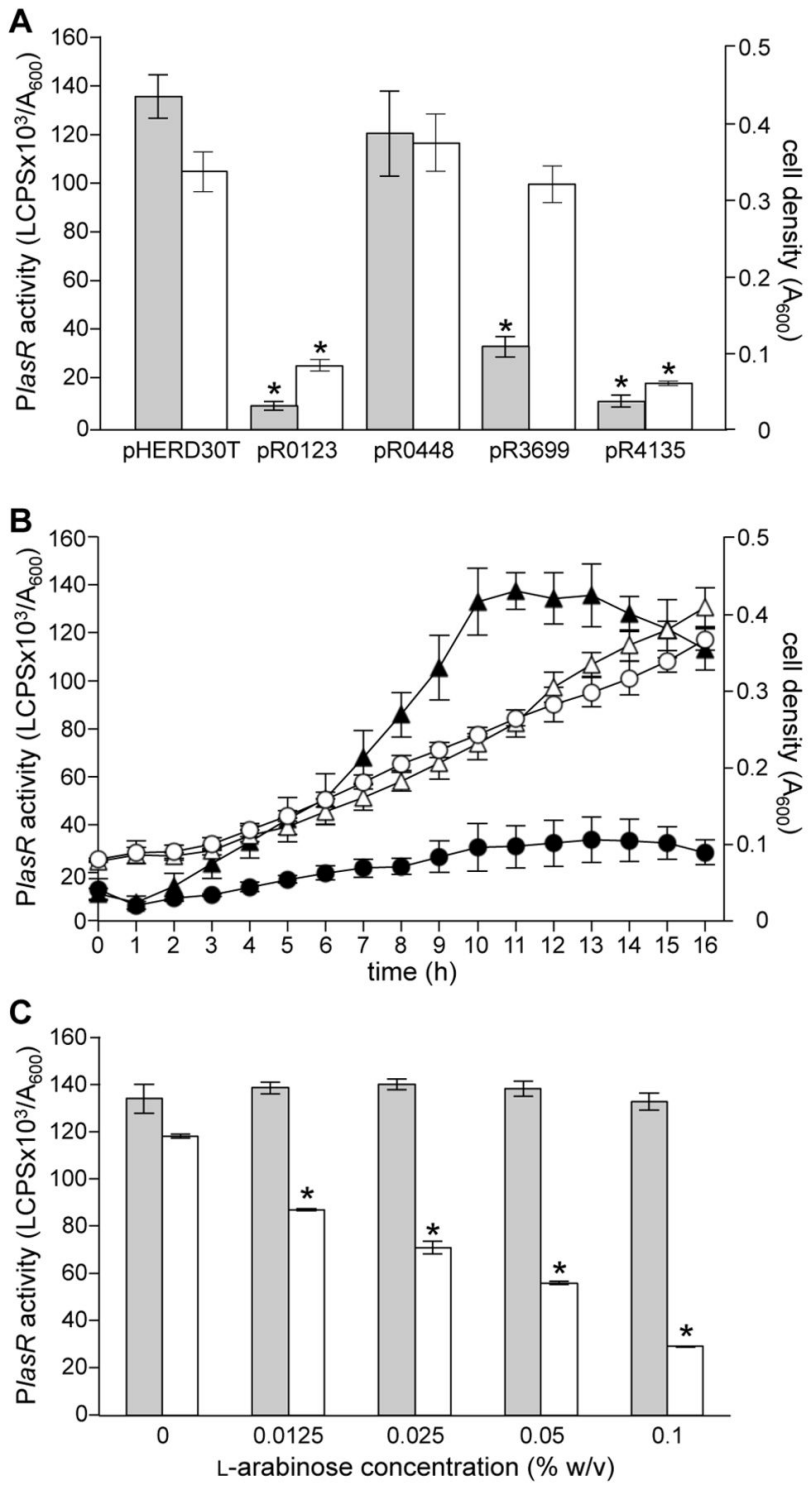
Effect of the induction of P*lasR*-bound proteins on P*lasR* promoter activity. (A) Histogram reporting P*lasR* maximal promoter activity (grey bars) and the corresponding cell density (white bars) measured in *P. aeruginosa* PAO1 P*lasR::lux* strains carrying the plasmids indicated below the graph, grown in LB supplemented with 0.1% (w/v) l-arabinose. (B) Graph reporting P*lasR* promoter activity (filled symbols) and cell density (open symbols) measured during the growth curve in *P. aeruginosa* PAO1 P*lasR::lux* carrying pHERD30T (triangles) or pR3699 (circles), grown in LB supplemented with 0.1% (w/v) l-arabinose. (C) Histogram reporting P*lasR* maximal promoter activity measured in *P. aeruginosa* PAO1 P*lasR::lux* strains carrying pHERD30T (grey bars) or pR3699 (white bars) grown in LB supplemented with different l-arabinose concentrations (%, w/v), indicated below the graph. In (A), (B) and (C) the average of three independent experiments is reported with standard deviations; in (A) and (C) statistical significance with respect to *P. aeruginosa* PAO1 P*lasR::lux* (pHERD30T) is indicated with one asterisk (*p* < 0.01).

It was surprising that inactivation of the four new putative regulators able to bind P*lasR in vitro* did not affect the activity of this promoter *in vivo*. However, the DNA-affinity chromatography allows the purification of proteins present in a synthetic binding buffer, a condition that is unlikely to mimic the intracellular milieu. Thus, some of the factors picked-up *in vitro* using P*lasR* as bait could be not sufficiently expressed and/or active *in vivo* under the experimental conditions used for the promoter activity assay. To overcome this problem, the PA0123, PA0448, PA3699, and PA4135 genes were cloned in the expression vector pHERD30T, under the control of an l-arabinose-inducible promoter (Table S2) [23]. The resulting plasmids were independently introduced in the wild type *P. aeruginosa* PAO1 strain carrying the P*lasR*::*lux* transcriptional fusion, and promoter activity was measured along the growth curve in the presence of l-arabinose; the pHERD30T empty vector was used as control. With the only exception of PA0448, l-arabinose-induced expression of the tested proteins reduced the activity of the P*lasR*::*lux* fusion. However, induction of PA0123 and PA4135 also resulted in a strong inhibition of *P. aeruginosa* growth (Figure 2A). Since bioluminescence emission is an energy demanding process, we hypothesized that PA0123 and PA4135 induction might affect growth only in a *lux* proficient background. However, PA0123 and PA4135 induction had a negative effect on *P. aeruginosa* growth also in the PAO1 wild type strain lacking the P*lasR*::*lux* fusion in the chromosome (data not shown). Overall, these results indicate that PA0123 and PA4135 are toxic for *P. aeruginosa* when their expression is induced, at least in the growth conditions used in our experimental setting.

Since an effect on bacterial growth is particularly meaningful when studying a cell density-dependent pathway like QS, further studies were focused on PA3699, the only protein that, once induced, strongly inhibited P*lasR* activity without affecting *P. aeruginosa* growth (Figure 2A).

It could be reasonably argued that the procedure described in the above paragraphs could isolate DNA binding proteins that do not play a role *in vivo* on the regulation of the target promoter and that the overexpression of transcriptional regulators could lead to pleiotropic effects/phenotypes. These causalities likely occurred in the case of PA0448, PA0123 and PA4135, since the first protein has no effect on P*lasR* activity and the other two are toxic to the cell when overexpressed. Conversely, PA3699 induction did not affect growth, while it repressed P*lasR* activity during the whole growth curve and proportionally to the amount of l-arabinose present in the growth medium (Figure 2B and 2C). All these features argue for a *bona fide* repressor activity of PA3699 on P*lasR*.

### PA3699 binds the *lasR* promoter region in EMSA assays

**Figure 3:**
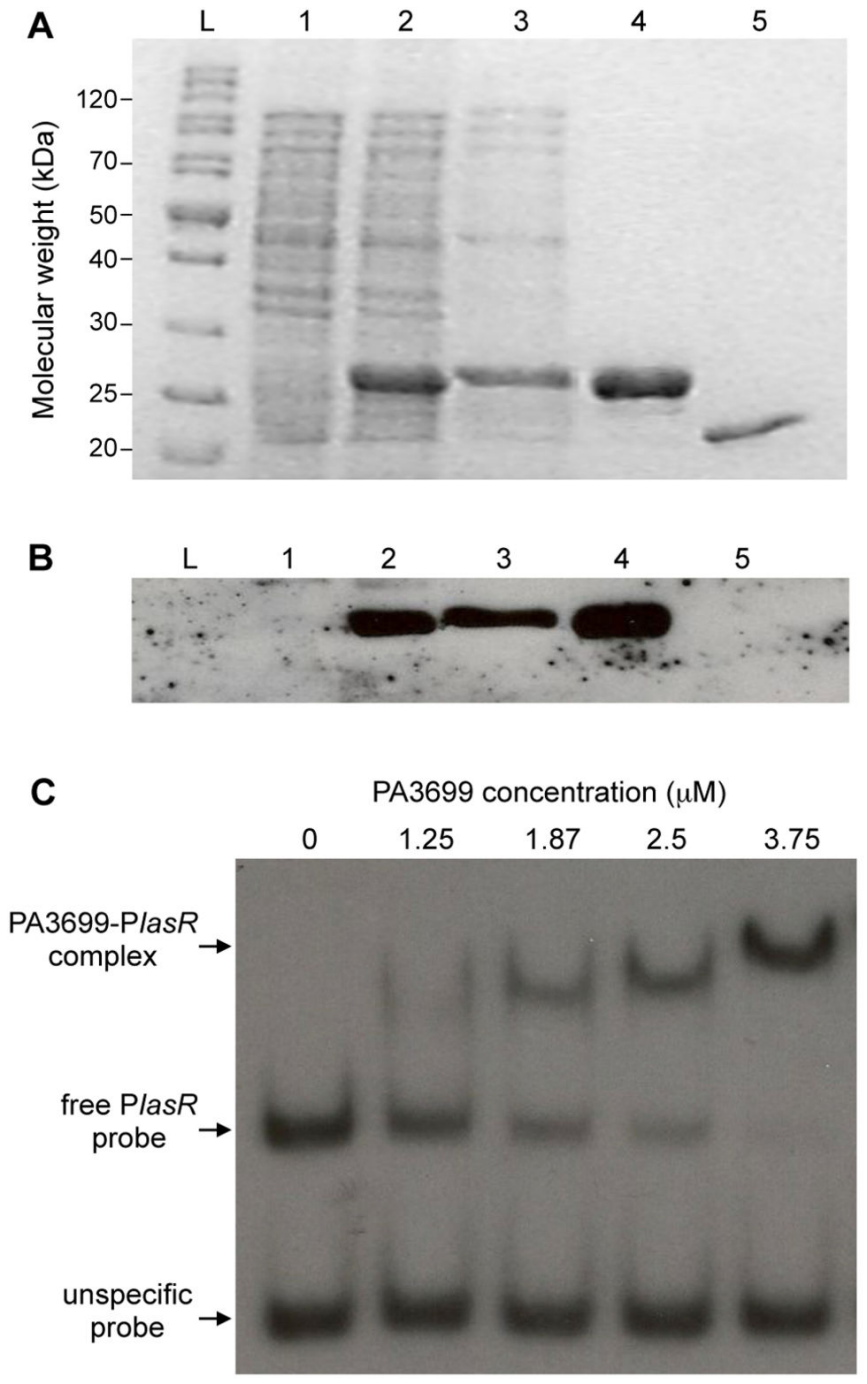
PA3699 purification and P*lasR*-binding assay. (A) SDS-PAGE analysis of samples withdrawn at different steps of PA3699 purification. Lane L, PageRuler Unstained Protein Ladder (Fermentas); lane 1, non-induced protein crude extract; lane 2, induced protein crude extract; lane 3, soluble fraction of the induced protein crude extract; lane 4, purified protein; line 5, purified protein after thrombin cleavage. (B) Western blot analysis performed with mouse anti-6xHis primary antibody and anti-mouse peroxidase-conjugated secondary antibody on a gel identical to the one shown in (A). (C) Autoradiography of an EMSA showing direct interaction between a DNA probe encompassing the *lasR* promoter region and purified PA3699. PA3699 concentration (μM) is indicated above each lane. An unspecific probe was added in the reaction mixture as control. The PA3699-P*lasR* complex and the free DNA probes are indicated.

In order to confirm DNA-affinity chromatography results, electrophoretic mobility shift assay (EMSA) of a DNA probe (P*lasRp*) encompassing the promoter region of *lasR* were conducted using the purified PA3699 protein. To this purpose, PA3699 was fused to a six-histidine tag (6xHis) at the N-terminal domain and purified by nickel-nitrilotriacetic acid (Ni-NTA)-affinity chromatography. The 6×His tag was subsequently cleaved by thrombin digestion, yielding the native PA3699 protein in soluble and highly pure form (Figure 3A and 3B).

As shown in Figure 3C, incubation of the P*lasRp* probe with purified PA3699 led to the formation of a complex endowed with lower electrophoretic motility with respect to the free probe, and the amount of the shifted complex was proportional to the concentration of purified PA3699. Since the electrophoretic motility of an unspecific DNA probe did not change in the presence of PA3699, the binding of this protein to P*lasRp* is specific (Figure 3C).

Altogether, these data provide an unequivocal proof of the direct molecular interaction between PA3699 and the *lasR* promoter region.

### PA3699 affects the production of QS-controlled virulence factors

The LasR/3OC_12_-HSL complex is required for full expression of most *P. aeruginosa* virulence factors, including elastase, pyocyanin, and proteases [24].

**Figure 4:**
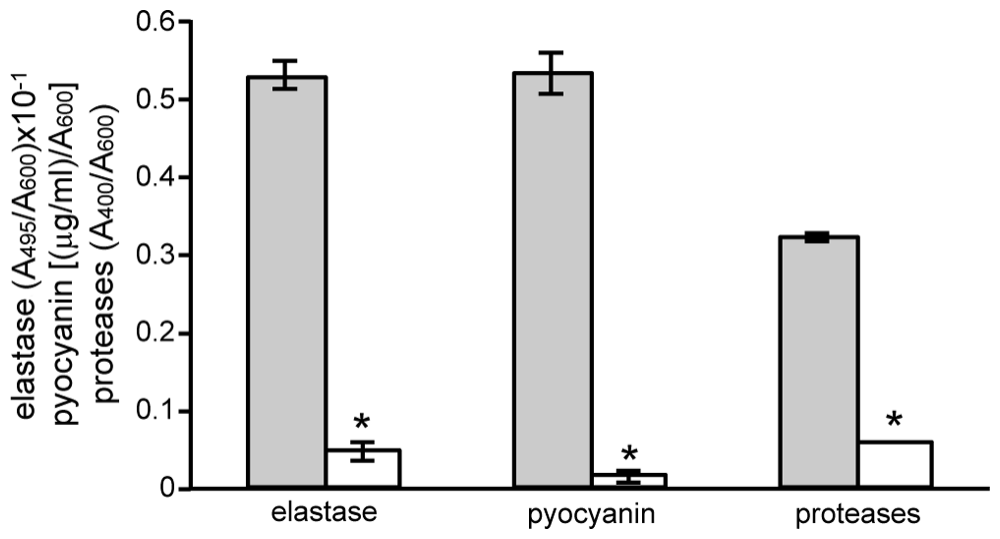
Effect of PA3699 induction on *P. aeruginosa* virulence factors production. Histogram reporting elastase, pyocyanin and proteases production measured in *P. aeruginosa* PAO1 carrying pHERD30T (grey bars) or pR3699 (white bars), grown in LB supplemented with 0.1% (w/v) l-arabinose. The average of three independent experiments is reported with standard deviations; statistical significance with respect to *P*. *aeruginosa* PAO1 (pHERD30T) is indicated with one asterisk (*p* < 0.01).

In order to assess whether the repression exerted by PA3699 on LasR expression may affect *P. aeruginosa* pathogenic potential, the expression of LasR-dependent virulence phenotypes was compared in *P. aeruginosa* carrying either the pHERD30T empty vector or its derivative plasmid for l-arabinose-dependent induction of PA3699 (pR3699). Consistent with the strong repressive effect exerted by PA3699 induction on P*lasR* activity, results showed that also the production of LasR-dependent phenotypes such as elastase, pyocyanin, and proteases was strongly decreased in *P. aeruginosa* upon PA3699 induction (Figure 4). However, pyocyanin, elastase and protease production remained unaffected in the *P. aeruginosa* PA3699 mutant strain with respect to the wild type, consistently with what observed for the P*lasR* promoter activity (data not shown).

## CONCLUDING REMARKS

The masterly management of an enormous metabolic potential *via* multiple regulative networks and signalling systems allows *P. aeruginosa* adaptation to the most challenging environments, including the human host [9,11,12].

The *P. aeruginosa las* QS system is among the most studied and best characterized bacterial regulatory pathways, also because it is considered a promising target for anti-virulence therapies [25–27]. Nevertheless, its interconnections with other *P. aeruginosa* regulative networks and signalling systems are still poorly understood [9].

So far, *in vivo* (genetic) approaches led to the identification of a number of genes involved in the modulation of the *las* system activity/response, highlighting the multiplicity of pathways connecting cell-density and environmental/metabolic signalling. However, the transcriptional factors directly controlling the expression of the *las* genes in response to environmental/metabolic stimuli are mostly unknown [11,12,15]. In this work, DNA-affinity chromatography has been used for the first time for the identification of transcriptional regulators of the *lasR* gene, encoding the 3OC_12_-HSL receptor.

Unexpectedly, the genetic inactivation of the four putative P*lasR* transcription factors identified by *in vitro* DNA-affinity chromatography (i.e., PA0123, PA0448, PA3699, and PA4135) did not affect the activity of P*lasR in vivo*. A possible explanation for this result is that at least some of these factors are not sufficiently expressed and/or active *in vivo* under the conditions used in the promoter activity assay. This hypothesis is supported by the observation that a progressive increase of PA3699 induction caused a parallel decrease in P*lasR* activity, indicating this factor as a novel transcriptional repressor of P*lasR*.

According to its structural features, PA3699 belongs to the TetR-family, a preeminent group of transcriptional regulators in bacteria [28]. Transcriptional regulators of the TetR-family typically activate upon binding of a specific ligand to an allosteric site [28]. Thus, it is likely that PA3699 regulates *lasR* transcription only in the presence of a specific ligand when expressed at a physiological level. Since the interaction of a transcriptional regulator with its DNA target sequence is governed by a thermodynamic equilibrium between the bound and unbound form, the induction of PA3699 expression could shift this equilibrium to the bound state, resulting in P*lasR* regulation also in the absence of the actual stimulus/ligand. Phenotypic microarray experiments are in progress to identify the growth conditions leading to PA3699 full activity and/or expression.

*P. aeruginosa* genome encodes at least 38 TetR-family proteins, and PA3699 is the eighth member of this group functionally characterized so far and the first one shown to bind the *lasR* promoter *in vitro* [28–33].

Overall, DNA-affinity chromatography combined with induction of selected factors has been proven as a useful approach for the identification of regulators that are not highly expressed or active under standard laboratory conditions, but that might be relevant in specific environmental niches, like for instance infection sites.

Although further studies are required to assess the involvement of PA3699 in *P. aeruginosa* physiology and pathogenesis, this work represents a further step toward the disclosure of the complex network controlling cell-cell communication, social behaviours and host adaptability of *P. aeruginosa*, and hopefully toward the identification of new promising targets for future drug-research programmes.

## Materials and Methods

### Bacterial strains and media

The bacterial strains used in this study are listed in Table S1 (Supporting Information). All *E. coli* and *P. aeruginosa* strains were routinely grown at 37°C in Luria-Bertani broth (LB), LB supplemented with 1.5% (w/v) agar, or Pseudomonas Isolation Agar (PIA) [34]. Unless otherwise stated, antibiotics were added at the following concentrations: *E. coli*, 20 µg/ml gentamicin (Gm), 100 µg/ml ampicillin (Ap), 25 µg/ml kanamycin (Km), 30 µg/ml chloramphenicol (Cm), 10 µg/ml tetracycline (Tc), 10 µg/ml nalidixic acid (Nal); *P. aeruginosa*, 100 µg/ml Gm, 400 µg/ml Cm, 100 µg/ml Tc, 300 µg/ml carbenicillin (Cb). When required, media were supplemented with glucose, isopropyl β-D-1-thiogalactopyranoside (IPTG) or l-arabinose at the concentration reported in the text.

### Recombinant DNA techniques

Plasmids used or generated in this study and details on their construction are reported in Table S2 (Supporting Information). Preparation of plasmid DNA, purification of DNA fragments, restrictions, ligations, and transformations of *E. coli* were carried out by standard procedures [34]. PCR amplifications were performed using Bio Red-Taq DNA polymerase (Bioline) or Accuzyme DNA polymerase (Bioline). The oligonucleotides used in this study are listed in Table S3 (Supporting Information). Automated sequencing was performed by Genechron sequence service (Genechron).

When required, plasmids were transferred from *E. coli* S17.1 λ*pir* to *P. aeruginosa* PAO1 by bi-parental conjugation, and from *E. coli* DH5α to *P. aeruginosa* PAO1 by tri-parental conjugation with the helper strain *E. coli* HB101 pRK2013 [35].

### DNA-affinity chromatography

DNA-affinity purification was performed as previously described [36]. A biotinylated DNA fragment encompassing the *lasR* promoter region (from nucleotide -359 to +13 relative to the *lasR* start codon) was PCR amplified with primers FW381 and RV334 (Table S3); primer FW381 was biotinylated.

*P. aeruginosa* PAO1 protein crude extracts were obtained from cells grown in 300 ml of LB at 37°C to an absorbance at 600 nm wavelength (A_600_) of 2.0 or 5.0, with 200 r.p.m. shaking. Bacterial cells were harvested by centrifugation and suspended in 2 ml of Sonication Buffer [10 mM Tris-HCl pH 8.0, 100 mM NaCl, 1 mM ethylenediaminetetraacetic acid (EDTA), 0.05% (v/v) Triton X-100] supplemented with 1 mg/ml lysozyme. After 30 min of incubation at 37°C, cells were disrupted by sonication. Cellular debris were removed by centrifugation and subsequent filtration. Protein concentration in the supernatant was determined with the Bradford Protein Assay Kit (Bio-Rad) according to manufacturer’s instruction.

One mg of paramagnetic streptavidin-conjugated resin (Dynabeads M-280, Invitrogen) was equilibrated in 200 µl of Wash Buffer 2X [10 mM Tris-HCl pH 7.5, 1 mM EDTA, 2 M NaCl, 0.05% (v/v) Triton X-100], and then incubated with 20 µg of the biotin-labelled PCR product for 25 min at 22°C. Unbound DNA was removed by magnetic separation in a magnet particle concentrator (Dynal, Invitrogen). Dynabeads were washed three times with 1 ml Wash Buffer 1X, and then incubated with 50 mg of *P. aeruginosa* protein crude extract for 2 hours at 22°C. Unbound proteins were removed by magnetic separation in a magnet particle concentrator, and the Dynabeads were washed six times with Sonication Buffer. Proteins specifically bound to the DNA-bead complexes were eluted in 20 µl of Elution Buffer [1.2 M NaCl, 10 mM Tris-HCl pH 8.0, 1 mM EDTA, 0.05% (v/v) Triton X100]. Eluted proteins were separated by Sodium Dodecyl Sulphate PolyAcrylamide Gel Electrophoresis (SDS-PAGE) on a 12% (v/v) polyacrylamide gel and stained with Coomassie Brilliant Blue [34]. Protein identity was determined by Matrix-Assisted Laser Desorption/Ionization-Time Of Flight (MALDI-TOF) mass spectrometry. MALDI-TOF analyses were performed as previously described [37].

### Construction of *P. aeruginosa* mutants

*P. aeruginosa* in frame deletion mutants were generated using the pDM4 plasmid as previously described [38]. Briefly, the flanking DNA regions (about 650 bp each) of the selected genes were PCR amplified with primers listed in Table S3 and sequentially cloned in pDM4. The resulting pDM4-derivative plasmids, listed in Table S2, were independently introduced in *P. aeruginosa* PAO1 by conjugation. Clones with a chromosomal insertion of the pDM4-derivative plasmids were selected on LB agar plates supplemented with 400 µg/ml Cm e 10 µg/ml Nal. Plasmid excision from the chromosome was subsequently selected on LB agar plates supplemented with 10% (w/v) sucrose. Mutant strains were checked by PCR analysis.

### Construction of *PlasR* transcriptional fusions

The DNA region encompassing the *lasR* promoter (spanning from nucleotide -359 to +13 relative to the *lasR* start codon) was PCR amplified with the primers FW333 and RV334 (Table S3) and cloned by EcoRI-PstI restriction in the pMP220 and mini-CTX-*lux* plasmids [39,40]. The resulting pMP*lasR*::*lacZ* and mini-CTX-P*lasR*::*lux* plasmids (Table S2) were conjugated in selected *P. aeruginosa* strains. The mini-CTX plasmid backbone was removed from the *P. aeruginosa* strains carrying the P*lasR::lux* cassette by using the pFLP2 plasmid (Table S2). pFLP2 was subsequently cured by sucrose counterselection as previously described [41].

### Over-expression of putative *lasR* regulators

The PA0123, PA0448, PA3699 and PA4135 genes were PCR amplified using the primers listed in Table S3 and cloned in the pHERD30T vector [23], generating the plasmids pR0123, pR0448, pR3699 and pR4135, respectively (Table S2). These plasmids were introduced in *P. aeruginosa* PAO1 carrying the P*lasR::lux* transcriptional fusion by conjugation. Positive clones were selected on PIA plates supplemented with 100 µg/ml Gm.

### Promoter activity assay

P*lasR* activity in *P. aeruginosa* PAO1 carrying the pMP*lasR*::*lacZ* plasmid was measured by standard Miller assay [42]. An over-night culture was diluted to an A_600_ of 0.05 in fresh LB, and incubated for 18 hrs at 37°C. Promoter activity (M.u.) and cell density (A_600_) were determined every 2 hr for 18 hrs.

P*lasR* activity in *P. aeruginosa* strains carrying the P*lasR*::*lux* chromosomally-inserted transcriptional fusion was determined as bioluminescence emission per cell by using the automated luminometer-spectrometer Wallac 1420 VICTOR 3V (PerkinElmer). Over-night cultures were diluted to an A_600_ of 0.05 in fresh LB, and 0.2 ml of these cultures were grown at 37°C in microtiter plates. Luminescence and cell density (A_600_) were determined every 1 hr for 16 hrs. Promoter activity is given as Light Counts Per Second (LCPS) divided by A_600_. The strains carrying the pHERD30T-derivative plasmids were grown in LB supplemented with Gm 100 µg/ml and 0.1% l-arabinose. For both the *lacZ*- and *lux*-based promoter assays average values and standard deviations were calculated from three independent experiments.

### PA3699 purification

The PA3699 gene was cloned in the pET-28b(+) vector (Novagen), generating the pE3699-N6 plasmid (for more details see Table S2). In this plasmid the PA3699 gene is transcriptionally-coupled in frame with a sequence coding for a 6 histidine tag (6xHis) at the N-terminus.

The pE3699-N6 plasmid was transformed in *E. coli* BL21 (DE3, pLysS) (Novagen). The resulting strain was grown over-night at 37°C with 200 r.p.m. shaking in LB supplemented with 25 µg/ml Km, 30 µg/ml Cm and 0.2% (w/v) glucose. The over-night culture was diluted 1:100 in 250 ml of the same medium and after 1 hr growth at 37°C with 200 r.p.m. shaking it was induced for 4 hrs with 0.5 mM IPTG.

PA3699-N6 purification was performed with the QIAexpress® Ni-NTA Fast Start Kit (Qiagen), according to manufacturer’s instructions. The PA3699-N6 recombinant protein was eluted from the Ni-NTA resin with 1 ml of Elution Buffer Native, dialyzed 1:1000 in Dialysis Buffer (20 mM Tris-HCl pH 7.5, 500 mM NaCl), and concentrated with Centricon YM-10 centrifugal filter device (Amicon). PA3699-N6 identity was verified by SDS-PAGE and Western blot analysis performed with anti-6xHis primary antibody (Qiagen) and peroxidase-conjugated anti-mouse IgG secondary antiboby (Sigma-Aldrich) on aliquots withdrawn at different steps of the purification process [34]. The 6×His tag was subsequently cleaved from 1 mg of purified PA3699-N6 by digestion with 100 µg thrombin in Thrombin Buffer (50 mM Tris-HCl pH 8.0, 150 mM NaCl, 3.3 mM CaCl_2_). The PA3699 protein was further purified by gel permeation chromatography on a Superdex 200 HR column (Amersham Pharmacia Biotech), dialyzed 1:1000 in Storage buffer (50 mM Tris-HCl pH 8.0, 150 mM NaCl, 50% glycerol) and stored at -20°C.

Protein concentration was determined with the Bradford protein assay kit (Bio-Rad) according to manufacturer’s instruction.

### Electrophoretic Mobility Shift Assay (EMSA)

A 210 bp DNA fragment encompassing the *lasR* promoter region (from nucleotides -359 to -150 relative to the *lasR* start codon) was PCR amplified with primers FW 333 and RV 535 (Table S3) from *P. aeruginosa* PAO1 genome and T/A cloned in the pDRIVE-T-EASY vector (Qiagen), generating pDP*lasR* (for more details see Table S2). pDP*lasR* was digested with EcoRI, and the resulting 218 bp DNA fragment was labeled with [α-^32^P] dATP by fill-in with Klenow enzyme [43]. The radio-labelled DNA probe was purified with SigmaSpin post-reaction purification columns (Sigma-Aldrich) followed by phenol-chloroform (1:1) extraction and ethanol precipitation.

In the EMSA experiments, the labelled DNA probe (0.2 nM) was incubated with different protein concentrations, in Binding Buffer [20 mM Tris-HCl, 2 mM EDTA, 5 mM MgCl_2_, 30 mM KCl, 5% (v/v) glycerol, 0.025% (v/v) nonidet P-40, 30 µg/ml poly(dI): (dC); pH 8.0]. A labelled aspecific probe (0.2 nM) was added to the binding mix. After 15 min of incubation at 22°C the reaction mixtures were loaded onto a 30 min pre-run 6% (w/v) polyacrylamide gel under non-denaturing conditions. The ratio acrylamide: bis-acrylamide was 37.5:1. The electrophoresis was carried out at 22°C in TBE 0.5X [34] at 5 V/cm for 4 hrs. Gel was then dried and autoradiographed.

### Phenotypic assays

*P. aeruginosa* PAO1 wild type carrying the pHERD30T empty vector or the pR3699 plasmid (see Table S2) were grown over night at 37°C in LB supplemented with 100 µg/ml Gm. Cultures were diluted to an A_600_ of 0.02 in LB supplemented with 0.1% l-arabinose, and supernatants were collected for elastolytic, proteolytic and pyocyanin assays after 6 hrs incubation at 37°C with 200 r.p.m shaking.

Elastolytic and proteolytic activities were determined by elastin-Congo red and azocasein assays, as previously described [44–46]. Pyocyanin quantification was performed as previously described [47]. The amount of pyocyanin, in μg/ml, was calculated using the following formula: A_520_/A_600_ X 17.072 = μg of pyocyanin per ml [48].

The average data and standard deviations of the phenotypic assays were calculated from three independent experiments.

### Statistical analysis

Statistical significance was determined by calculating the *p*-values using the two-tailed Student-t test for unpaired data sets; differences with a *p*-value ≤ 0.01 are considered as statistically significant.

## Supporting Information

Figure S1 Effect of the mutations in the genes coding for the new putative *lasR* transcriptional regulators on P*lasR* activity.
Graph reporting P*lasR* promoter activity measured in *P. aeruginosa* PAO1 wild type (black line) and in isogenic clear deletion mutants in the PA0123 (green line), PA0448 (red line), PA3699 (blue line), PA4135 (yellow line) or *vfr* (grey line) gene. Each strain contained the P*lasR*::*lux* transcriptional fusion in the chromosomal *attB* neutral site, and was grown for 16 hrs in LB at 37°C with 200 r.p.m. shaking. P*lasR* activity is given as Light Counts Per Second (LCPS) divided by cell density (A_600_). The average values and standard deviations were calculated from three independent experiments. Statistical significance with respect to the *P*. *aeruginosa* PAO1 wild type strain is indicated with one asterisk (*p* < 0.01).
(PDF)

Table S1 Bacterial strains used in this study.
(PDF)

Table S2 Plasmids used in this study.
(PDF)

Table S3 Oligonucleotides used in this study.
(PDF)
