# Supplementary material for: A New Transcriptional Repressor of the Pseudomonas aeruginosa Quorum Sensing Receptor Gene lasR
Source: PLoS One. 2013 Jul 5;8(7):e69554. doi: 10.1371/journal.pone.0069554 (PMC3702619; doi:10.1371/journal.pone.0069554)
Supplement: Figure S1 — Effect of the mutations in the genes coding for the new putative lasR transcriptional regulators on PlasR activity. Graph reporting PlasR promoter activity measured in P. aeruginosa PAO1 wild type (black line) and in isogenic clear deletion mutants in the PA0123 (green line), PA0448 (red line), PA3699 (blue line), PA4135 (yellow line) or vfr (grey line) gene. Each strain contained the PlasR::lux transcriptional fusion in the chromosomal attB neutral site, and was grown for 16 hrs in LB at 37°C with 200 r.p.m. shaking. PlasR activity is given as Light Counts Per Second (LCPS) divided by cell density (A600). The average values and standard deviations were calculated from three independent experiments. Statistical significance with respect to the P. aeruginosa PAO1 wild type strain is indicated with one asterisk (p < 0.01). (PDF) [file pone.0069554.s001.pdf]

# Supporting Information

# Figure S1

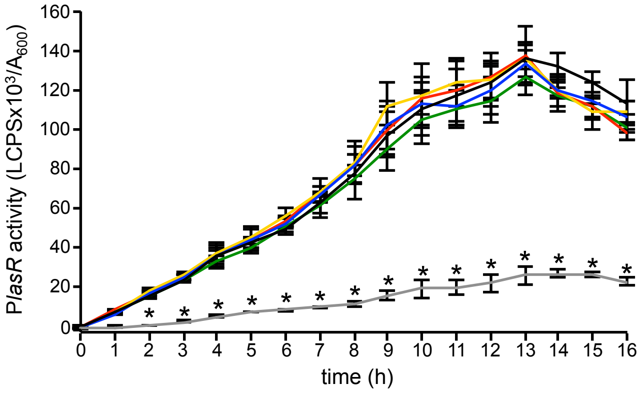


**Figure S1. Effect of the mutations in the genes coding for the new putative *lasR* transcriptional regulators on P*lasR* activity.**

Graph reporting P*lasR* promoter activity measured in *P. aeruginosa* PAO1 wild type (black line) and in isogenic clear deletion mutants in the PA0123 (green line), PA0448 (red line), PA3699 (blue line), PA4135 (yellow line) or *vfr* (grey line) gene. Each strain contained the P*lasR::lux* transcriptional fusion in the chromosomal *attB* neutral site, and was grown for 16 hrs in LB at 37°C with 200 r.p.m. shaking. P*lasR* activity is given as Light Counts Per Second (LCPS) divided by cell density ($A_{600}$). The average values and standard deviations were calculated from three independent experiments. Statistical significance with respect to the *P. aeruginosa* PAO1 wild type strain is indicated with one asterisk ($p < 0.01$).
