# Supplementary material for: A New Transcriptional Repressor of the Pseudomonas aeruginosa Quorum Sensing Receptor Gene lasR
Source: PLoS One. 2013 Jul 5;8(7):e69554. doi: 10.1371/journal.pone.0069554 (PMC3702619; doi:10.1371/journal.pone.0069554)
Supplement: Table S1 — Bacterial strains used in this study. (PDF) [file pone.0069554.s002.pdf]

## SUPPORTING INFORMATION

**Table S1.** Bacterial strains used in this study.

| **Strain** | **Relevant characteristics** | **Reference/Source** |
|---|---|---|
| ***E. coli*** | | |
| DH5α | Cloning strain; $Nal^R$. | Grant *et al.*, 1990 |
| S17.1 λ*pir* | Conjugative strain for suicide plasmids. | Simon *et al.*, 1983 |
| BL21 (DE3, pLysS) | High stringency expression host; $Cm^R$. | Novagen |
| HB101 (pRK2013) | Helper strain for tri-parental conjugations; $Km^R$. | Figursky and Helinski, 1979 |
| SM10 (pFLP2) | Strain carrying the pFLP2 plasmid (FLP; $Ap^R$/$Cb^R$) for Flp-mediated recombination. | Hoang *et al.*, 1998 |
| ***P. aeruginosa*** | | |
| PAO1 (ATCC15692) | wild type strain. | American Type Culture Collection |
| PAO1 P*lasR*::*lux* | wild type strain carrying the P*lasR*::*lux* transcriptional fusion integrated in the chromosome. | This study |
| ΔPA0123 | *P. aeruginosa* PAO1 mutant strain with a complete deletion of the PA0123 gene. | This study |
| ΔPA0123 P*lasR*::*lux* | ΔPA0123 carrying the P*lasR*::*lux* transcriptional fusion integrated in the chromosome. | This study |
| ΔPA0448 | *P. aeruginosa* PAO1 mutant strain with a complete deletion of the PA0448 gene. | This study |
| ΔPA0448 P*lasR*::*lux* | ΔPA0448 carrying the P*lasR*::*lux* transcriptional fusion integrated in the chromosome. | This study |
| ΔPA3699 | *P. aeruginosa* PAO1 mutant strain with a complete deletion of the PA3699 gene. | This study |
| ΔPA3699 P*lasR*::*lux* | ΔPA3699 carrying the P*lasR*::*lux* transcriptional fusion integrated in the chromosome. | This study |
| ΔPA4135 | *P. aeruginosa* PAO1 mutant strain with a complete deletion of the PA4135 gene. | This study |
| ΔPA4135 P*lasR*::*lux* | ΔPA4135 carrying the P*lasR*::*lux* transcriptional fusion integrated in the chromosome. | This study |
| Δ*vfr* | *P. aeruginosa* PAO1 mutant strain with a complete deletion of the *vfr* gene. | This study |
| Δ*vfr* P*lasR*::*lux* | Δ*vfr* carrying the P*lasR*::*lux* transcriptional fusion integrated in the chromosome. | This study |


**References:**

- Figursky DH, Helinski DR (1979) Replication of an origin-containing derivative of plasmid RK2 dependent on a plasmid function provided in trans. *Proc Natl Acad Sci USA* 76: 1648-1652.
- Grant SG, Jessee J, Bloom FR, Hanahan D (1990) Differential plasmid rescue from transgenic mouse DNAs into *Escherichia coli* methylation-restriction mutants. *Proc Natl Acad Sci USA* 87: 4645-4649.
- Hoang TT, Karkhoff-Schweizer RR, Kutchma AJ, Schweizer HP (1998) A broadhost-range Flp-FRT recombination system for site-specific excision of chromosomally located DNA sequences: application for isolation of unmarked *Pseudomonas aeruginosa* mutants. Gene 212: 77-86.
- Simon R, Priefer U, Puhler A (1983) A broad host range mobilization system for *in vivo* genetic-engineering: transposon mutagenesis in Gram-negative bacteria. *Biotechnology* 1: 784-791.
