# Supplementary material for: A New Transcriptional Repressor of the Pseudomonas aeruginosa Quorum Sensing Receptor Gene lasR
Source: PLoS One. 2013 Jul 5;8(7):e69554. doi: 10.1371/journal.pone.0069554 (PMC3702619; doi:10.1371/journal.pone.0069554)
Supplement: Table S2 — Plasmids used in this study. (PDF) [file pone.0069554.s003.pdf]

SUPPORTING INFORMATION

Table S2. Plasmids used in this study.

Plasmid	Relevant characteristics and plasmid construction	Reference Source
pDM4	Suicide vector for construction of deletion mutants; *sacBR*; *oriR6K*; Cm^R.	Milton *et al*., 1996
mini-CTX-*lux*	Promoter-probe vector containing the *luxCDABE* operon; Tc^R.	Becher and Schweizer, 2000
pHERD30T	Plasmid for L-arabinose-inducible protein expression in *P. aeruginosa*; Gm^R.	Qiu *et al*., 2008
pMP220	*lacZ*-based promoter probe vector; Tc^R.	Spaink *et al*., 1987
pET-28b(+)	Plasmid for recombinant protein expression in *E. coli*; Km^R.	Novagen
pDRIVE-T-EASY	*E. coli* T/A based cloning vector; Ap^R.	Qiagen
pDM4 ΔPA0123	The upstream DNA region and downstream DNA region of the PA0123 gene were PCR amplified from *P. aeruginosa* PAO1 genome using primers FW416/RV417 and FW418/RV419 (Tab. S3), and cloned in the pDM4 plasmid by XbaI-XhoI restriction.	This study
pDM4 ΔPA0448	The upstream DNA region and downstream DNA region of the PA0448 gene were PCR amplified from *P. aeruginosa* PAO1 genome using primers FW428/RV429 and FW430/RV431 (Tab. S3), and cloned in the pDM4 plasmid by XbaI-XhoI restriction.	This study
pDM4 ΔPA3699	The upstream DNA region and downstream DNA region of the PA3699 gene were PCR amplified from *P. aeruginosa* PAO1 genome using primers FW448/RV449 and FW450/RV451 (Tab. S3), and cloned in the pDM4 plasmid by XbaI-XhoI restriction.	This study
pDM4 ΔPA4135	The upstream DNA region and downstream DNA region of the PA4135 gene were PCR amplified from *P. aeruginosa* PAO1 genome using primers FW424/RV425 and FW426/RV427 (Tab. S3), and cloned in the pDM4 plasmid by XbaI-XhoI restriction.	This study
pDM4 Δ*vfr*	The upstream DNA region and downstream DNA region of the *vfr* gene were PCR amplified from *P. aeruginosa* PAO1 genome using primers FW456/RV457 and FW458/RV459 (Tab. S3), and cloned in the pDM4 plasmid by XbaI-SalI restriction.	This study
pMP*lasR*::*lacZ*	pMP220 derivative plasmid containing a 372 bp DNA fragment encompassing the *lasR* promoter region (P*lasR*). P*lasR* was PCR amplified from *P. aeruginosa* PAO1 genome using primers FW333 and RV334 (Tab. S3) and cloned in the pMP220 plasmid by EcoRI-PstI restriction.	This study
mini-CTX-P*lasR::lux*	mini-CTX-*lux* derivative plasmid containing a 372 bp DNA fragment encompassing the *lasR* promoter region (P*lasR*). P*lasR* was PCR amplified from *P. aeruginosa* PAO1 genome using primers FW333 and RV334 (Tab. S3) and cloned in the mini-CTX-*lux* plasmid by EcoRI-PstI restriction.	This study
pR0123	The PA0123 gene was PCR amplified from *P. aeruginosa* PAO1 genome using primers FW479 and RV480 (Tab. S3) and cloned in pHERD by NcoI-XbaI restriction. This plasmid allows the over-expression of PA0123 in *P. aeruginosa*.	This study

pR0448	The PA0448 gene was PCR amplified from *P. aeruginosa* PAO1 genome using primers FW485 and RV486 (Tab. S3) and cloned in pHERD by NcoI-PstI restriction. This plasmid allows the over-expression of PA0448 in *P. aeruginosa*.	This study
pR3699	The PA3699 gene was PCR amplified from *P. aeruginosa* PAO1 genome using primers FW495 and RV496 (Tab. S3) and cloned in pHERD by NcoI-XbaI restriction. This plasmid allows the over-expression of PA3699 in *P. aeruginosa*.	This study
pR4135	The PA4135 gene was PCR amplified from *P. aeruginosa* PAO1 genome using primers FW483 and RV484 (Tab. S3) and cloned in pHERD by NcoI-PstI restriction. This plasmid allows the over-expression of PA4135 in *P. aeruginosa*.	This study
pE3699-N6	The PA3699 gene was PCR amplified from *P. aeruginosa* PAO1 genome using primers FW514 and RV515 (Tab. S3) and cloned in pET-28b(+) by NdeI-XhoI restriction. This plasmid allows the over-expression of PA3699 fused with a 6xHis at the *N*-terminus.	This study
pDP*lasR*	The P*lasR* promoter region was PCR amplified from *P. aeruginosa* PAO1 genome using primers FW333 and RV535 (Tab. S3) and cloned in pDRIVE-T-EASY by T/A cloning. This plasmid was generated to label the probe encompassing the P*lasR* promoter.	This study

References:

- Becher A, Schweizer HP (2000) Integration-proficient *Pseudomonas aeruginosa* vectors for isolation of single-copy chromosomal *lacZ* and *lux* gene fusions. Biotechniques 29: 948-950.
- Milton DL, O'Toole R, Horstedt P, Wolf-Watz H (1996) Flagellin A is essential for the virulence of *Vibrio anguillarum*. J Bacteriol 178: 1310-1319.
- Qiu D, Damron FH, Mima T, Schweizer HP, Yu HD (2008) PBAD-based shuttle vectors for functional analysis of toxic and highly regulated genes in *Pseudomonas* and *Burkholderia* spp. and other bacteria. Appl Environ Microbiol 74: 7422-7426.
- Spaink HP, Okker RJH, Wijffelman CA, Pees E, Lugtenberg BJJ (1987) Promoters in the nodulation region of the *Rhizobium leguminosarum* Sym plasmid pRL1J1. Plant Mol Biol 9: 27-39.
