# Supplementary material for: A New Transcriptional Repressor of the Pseudomonas aeruginosa Quorum Sensing Receptor Gene lasR
Source: PLoS One. 2013 Jul 5;8(7):e69554. doi: 10.1371/journal.pone.0069554 (PMC3702619; doi:10.1371/journal.pone.0069554)
Supplement: Table S3 — Oligonucleotides used in this study. (PDF) [file pone.0069554.s004.pdf]

SUPPORTING INFORMATION

Table S3. Oligonucleotides used in this study.

Name	Sequence (5′-3′) [a]	Position [b]	Restriction site
FW333	NNNCCGGAATTCGGTCGCACGCGTGGCG	1557809	EcoRI
RV334	NNAAAACTGCAGCAACCAAGGCCATAGCGCT	1558182	PstI
FW381	Biotin-GGTCGCACGCGTGGCG	1557809	-
FW416	NNNGCTCTAGAATCGATGAAGGCTTGGCCG	143925	XbaI
RV417	NNCCGGAATTCCGCTGGAGCTGACCGAC	143256	EcoRI
FW418	NNCCGGAATTCCGCTGGAGCTGACCGAC	142370	EcoRI
RV419	NNCCGCTCGAGAACGCCGGAGGGCCTGA	141721	XhoI
FW424	NNNGCTCTAGATTCTCGCCTATGGGCGGC	4625678	XbaI
RV425	NNNAACTGCAGTGGCGTTGACATAGGGTCC	4624992	PstI
FW426	NNNAACTGCAGATAAAGCCCTGAAGCGAC	4624591	PstI
RV427	NNCCGCTCGAGGTCGAGAAGATGCACAGC	4623928	XhoI
FW428	NNNGCTCTAGAAGGCCGAAGCAGCCGATG	503485	XbaI
RV429	NNNAACTGCAGGATCTTGCGTCGCATGGA	504135	PstI
FW430	NNNAACTGCAGGGGCTGGGCTGAACGCG	505018	PstI
RV431	NNCCGCTCGAGTCGCTCTACTGGCTGCTCA	505661	XhoI
FW448	NNNGCTCTAGACCCAAGGTCCTCGGCCACG	4141323	XbaI
RV449	NNNCGGAATTCTACTCGGGACACGGTGTTT	4140661	EcoRI
FW450	NNNCGGAATTCATGGGCCTGTGACCTGA	4139970	EcoRI
RV451	NNCCGCTCGAGATCTCATTGGGTGACCCGC	4139308	XhoI
FW456	NNNGCTCTAGATCTCCACCCCGCCGCG	707316	XbaI
RV457	NNNCGGAATTCAATAGCTACCATGCCCGAGT	706661	EcoRI
FW458	NNNCGGAATTCGGCACCCGCTGAACAG	706024	EcoRI
RV459	NACGCGTCGACCGGAGATCGCCCGCAGCT	705365	SalI
FW479	NCATGCCATGGATTCGCTGAAAGGC	143246	NcoI
RV480	NNNGCTCTAGATCGGTCAGCTCCAGCG	142352	XbaI
FW483	NCATGCCATGGCAACGCCAAGACCCTCCC	4624980	NcoI
RV484	NAAAACTGCAGGTCGCTTCAGGGCTTTATCT	4624575	PstI
FW485	NCATGCCATGGGACGCAAGATCCCTTCCAC	504124	NcoI
RV486	NAAAACTGCAGGCGTTCAGCCCAGCCCG	505015	PstI
FW495	NCATGCCATGGCCCGAGTAGCTCCCAGAG	4140649	NcoI
RV496	NNNGCTCTAGATCAGGTCACAGGCCCATGA	4139953	XbaI
FW514	GGAATTCCATATGTCCCGAGTAGCTCCCA	4140651	NdeI
RV515	NNCCGCTCGAGTCACAGGCCCATGACCA	4139957	XhoI
FW535	CCCACTCTATAGAGTTGGCG	1558001	-

[a.] Introduced restriction sites are underlined.
[b.] Position with respect to the *Pseudomonas* Genome Database (www.pseudomonas.com).
